# Comparative Cytogenetics and Fluorescent Chromosome Banding in Five Indian Species of *Dipcadi* Medik

**DOI:** 10.3390/plants12132534

**Published:** 2023-07-03

**Authors:** Tundra Samanta, Timir B. Jha, Sudipta Ray, Sumita Jha

**Affiliations:** 1 Department of Botany, Calcutta University, 35, Ballygunge Circular Road, Kolkata 700019, India; ts90.bot@gmail.com (T.S.); srbot@caluniv.ac.in (S.R.); 2 Department of Botany, Maulana Azad College, Kolkata 700013, India; tbjha2000@yahoo.co.in

**Keywords:** *Dipcadi*, karyotype, fluorochrome banding, DAPI bands, CMA bands

## Abstract

The genus *Dipcadi* Medik. (Subfamily: Scilloideae) has a narrow distribution in India and several overlapping morphological traits make the genus taxonomically challenging at the species level. Cytogenetic characterization can provide additional taxonomic data and can be used to evaluate genetic diversity at the species level. We have accomplished comparative karyotype analysis and fluorescence banding patterns using 4′-6-Diamidino-2-phenylindole (DAPI) and Chromomycin A_3_ (CMA) in five Indian species for the first time. The karyotypes of *D. concanense* and *D. goaense* exhibited similar fluorochrome banding profiles. However, *D. montanum*, *D. ursulae and D. erythraeum* differ distinctly in their karyotypes. In all taxa, CMA^+ve^/DAPI^−ve^ or DAPI^0^ (GC-rich) constitutive heterochromatin was located at the constriction region or terminal satellite of the nucleolar chromosome. DAPI^+ve^/CMA^−ve^ or CMA^0^ (AT-rich) heterochromatin dominates in *D. montanum, D. ursulae and D. erythraeum*. However, *D. erythraeum* shows a distinct variation in fluorochrome banding pattern from all other species. The distribution of CMA and DAPI bands is a reflection of heterochromatin composition and variations acquired by different species. This characterization can be used to assess phylogenetic relationships in the understudied genus *Dipcadi* and may serve as a basis for other genomic analyses and evolutionary studies.

## 1. Introduction

The subfamily Scilloideae (family Asparagaceae) *sensu* Angiosperm Phylogeny Group, (APG III) [1] is a major group of small perennial bulbous plants, consisting of four monophyletic tribes: Hyacintheae, Ornithogaleae, Urgineeae and Oziroeeae [2,3,4,5]. Bulbous geophytes of this subfamily have long been used in traditional medicine and specialized metabolites from members of each tribe have been reported such as homoisoflavonoids and triterpenoids from Hyacintheae, bufadienolides from Urgineeae and cardenolides from Ornithogaleae [6,7,8,9,10,11]. Scilloideae is represented mainly by three genera in India viz. *Drimia* Jacq. ex Willd. (Urgineeae), *Dipcadi* Medik. (Ornithogaleae) and *Ledebouria* Roth (Hyacintheaee). Due to taxonomic disputes at interspecific levels [12], the three genera have been subjected to revision from time to time [2,3,4].

The genus *Dipcadi* Medik. is morphologically distinct from other genera in having tubular flowers, quadrate capsules and large discoid seeds [13]. There are confusing reports on the number of valid species of *Dipcadi* in India [12,14,15,16], ranging from seven to nine species [12,14,16,17]. Most of the species of *Dipcadi* in India are endemic to the Western Ghats, a biodiversity hotspot and a world heritage site [12,17]. Some species are assessed as threatened according to the red data book of Indian plants of which *D. concanense* and *D. reidii* were declared extinct but rediscovered and assessed as critically endangered [17,18]. *Dipcadi goaense* was located along the lateritic gravelly area of South Goa [19] and the species is known by a single population restricted to the type locality [14]. *Dipcadi erythraeum* is endemic to desert areas of Rajasthan. These species have several uniform and overlapping morphological characters, making the genus taxonomically difficult at the species level [12,20] and necessitating the study of additional parameters for the thorough characterization of taxa. The importance of the Indian species of *Dipcadi* resides not only in their endemism and narrow distribution but also in their phytochemical constituents [12,15,21,22,23,24].

Detailed cytogenetic and molecular phylogenetic studies are reported to be useful for the advancement of classification at the species level [2,3,25]. Globally, chromosome counts have been reported for 14 species of *Dipcadi* [25] showing wide diversity in chromosome number [26]. Among the species occurring in India, *D. concanense, D. goaense* and *D. saxorum* show 2n = 12 chromosomes while *D. montanum* and *D. ursulae* show 2n = 20 chromosomes [14,27,28,29]. *Dipcadi erythraeum* is reported to have 2n = 20 [30] as well as 2n = 22 chromosomes [31]. Meiosis shows regular bivalent formation in most of the species studied [14,30,31,32]. Karyotype analysis in the Indian species of *Dipcadi* exhibits asymmetric bimodal karyotypes but detailed characterization is lacking [14,30,31,32,33].

Over the years, molecular cytogenetics has rejuvenated research on plant chromosomes. Chromosomes prepared through the enzymatic maceration and air drying (EMA) method followed by Giemsa staining [34,35] provides distinct chromosomal morphology in a cytoplasm-free background. Application of contrasting base-specific fluorochrome dyes 4′-6-Diamidino-2-phenylindole (DAPI) and Chromomycin A_3_ (CMA) helps to identify heterochromatin blocks of repetitive DNA sequences directly on the chromosomes [36]. The present study is a continuation of our previous work on the karyological relationship and molecular phylogeny of the Indian taxa of Scilloideae [37,38]. It is evident that chromosomal evaluation and molecular phylogenetic study may complement each other to enrich existing knowledge regarding the relationship among different species of the understudied genus *Dipcadi*.

The objective of the present study was to establish fluorescent karyotypes of Indian species of *Dipcadi*, to evaluate the genetic diversity at chromosomal level in the species collected. Traditional taxonomic parameters such as vegetative and floral morphology, anatomy and pollen architecture have been reported to show very little or continuous variation in *Dipcadi* species and hence the study of additional parameters seems necessary. Molecular cytogenetic characterization providing precise knowledge on chromosome number and architecture is reported to be useful for phylogenetic studies and the advancement of classification at the species level. This characterization can be used to assess genetic diversity, provide additional taxonomic data, and serve as a basis for other genomic analyses and evolutionary studies.

## 2. Results

### 2.1. Chromosome Number and Karyotype Analysis

This karyo-morphometric analysis of the Indian species of *Dipcadi* is based on five species collected from Western Ghats (*D. concanense, D. goaense, D. montanum, D. ursulae*) and Rajasthan (*D. erythraeum*). Chromosome counts from 20–25 well-scattered metaphase plates of each population of each species (Table 1) revealed interspecific differences in the diploid chromosome number. *Dipcadi concanense* (a threatened species) and *D. goaense* (an endemic species) showed 2n = 12 chromosomes, while *D. montanum* and *D. ursulae* show 2n = 20 chromosomes and *D. erythraeum*, an endemic species from Rajasthan, showed 2n = 22 chromosomes.

**Figure 1:**
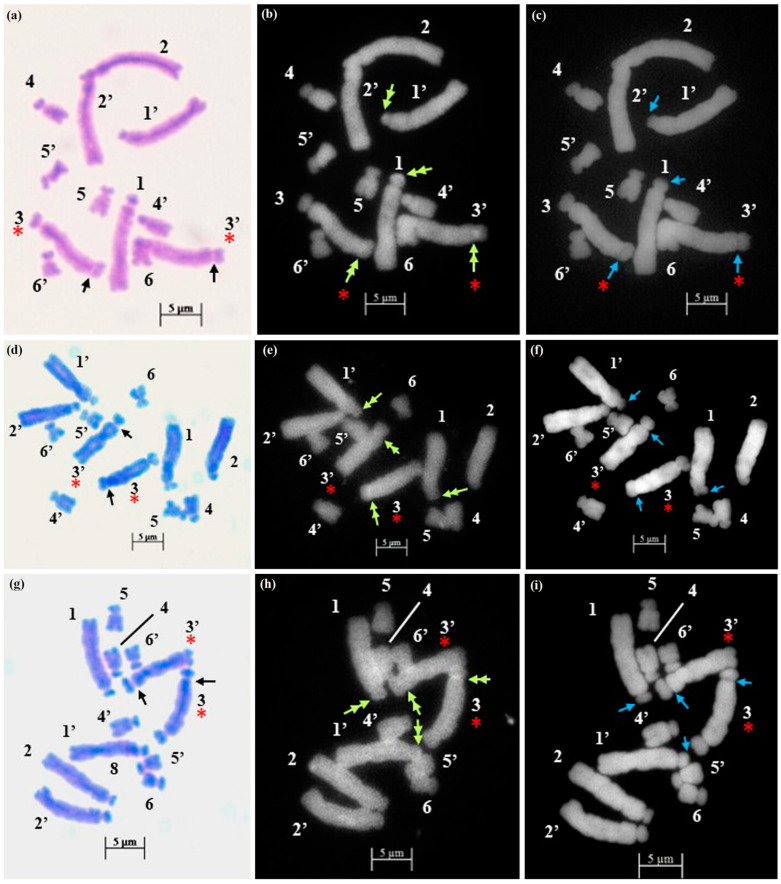
Somatic metaphase plates of *Dipcadi concanense* (Dalzell) Baker with 2n = 12 chromosomes stained with Giemsa (**a**,**d**,**g**), CMA (**b**,**e**,**h**) and DAPI (**c**,**f**,**i**)**.** Black arrows indicate position of secondary constrictions in Giemsa-stained plates (**a**,**d**,**g**). Double yellow arrows indicate CMA^+ve^ bands (**b**,**e**,**h**). Single blue arrows indicate the DAPI^−ve^ band/gap (**c**,**f**,**i**). Red asterisks mark chromosomes with secondary constrictions in all the plates. Bars 5 µm.

**Figure 2:**
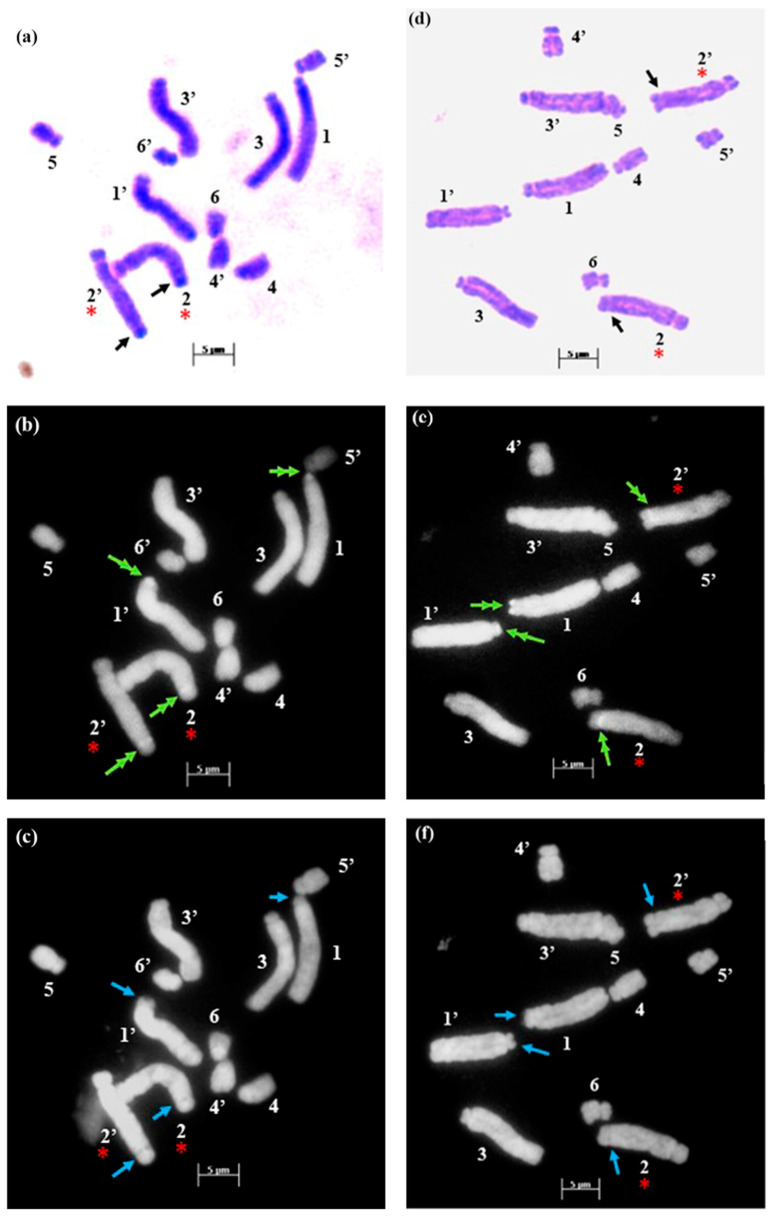
Somatic metaphase plates of *Dipcadi goaense* Prabhug. with 2n = 12 chromosomes stained with Giemsa (**a**,**d**), CMA (**b**,**e**) and DAPI (**c**,**f**). Black arrows indicate position of secondary constrictions in Giemsa-stained plates (**a**,**d**). Double yellow arrows indicate CMA^+ve^ bands (**b**,**e**). Single blue arrows mark the DAPI^−ve^ gaps (**c**,**f**). Red asterisks mark chromosomes with secondary constrictions in all the plates. (**d**–**f**); one chromosome less, out of field). Bars 5 µm.

**Figure 3:**
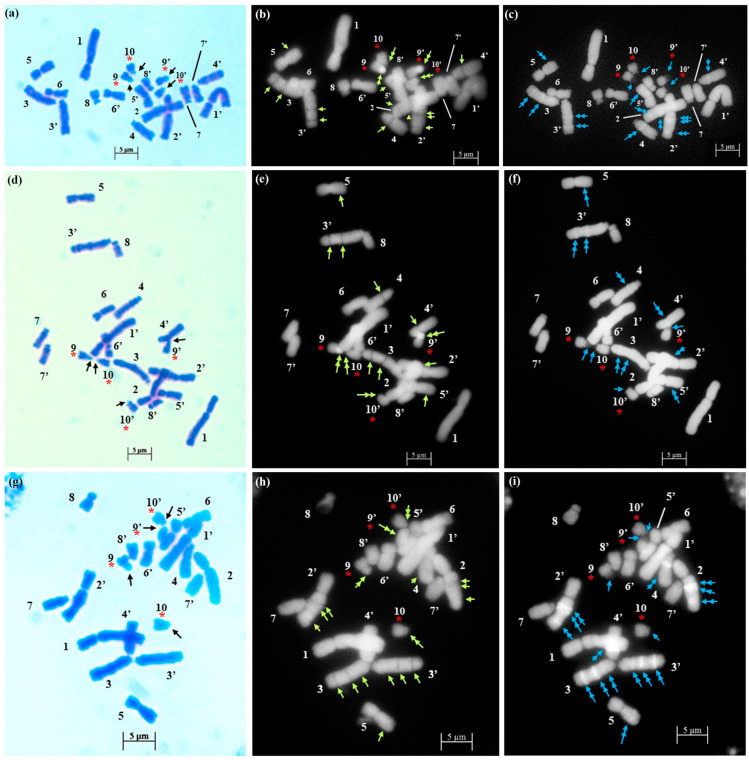
Somatic metaphase plates of *Dipcadi montanum* (Dalzell) Baker with 2n = 20 chromosomes stained with Giemsa (**a**,**d**,**g**), CMA (**b**,**e**,**h**) and DAPI (**c**,**f**,**i**)**.** Black arrows indicate position of secondary constrictions in Giemsa-stained plates (**a**,**d**,**g**). Double yellow arrows indicate CMA^+ve^ signals and single yellow arrows indicate CMA^−ve^ gaps (**b**,**e**,**h**). Double blue arrows indicate DAPI^+ve^ signals and single blue arrows mark the DAPI^−ve^ gaps (**c**,**f**,**i**). Red asterisks indicate chromosomes with secondary constrictions in all the plates. Bars 5 µm.

**Figure 4:**
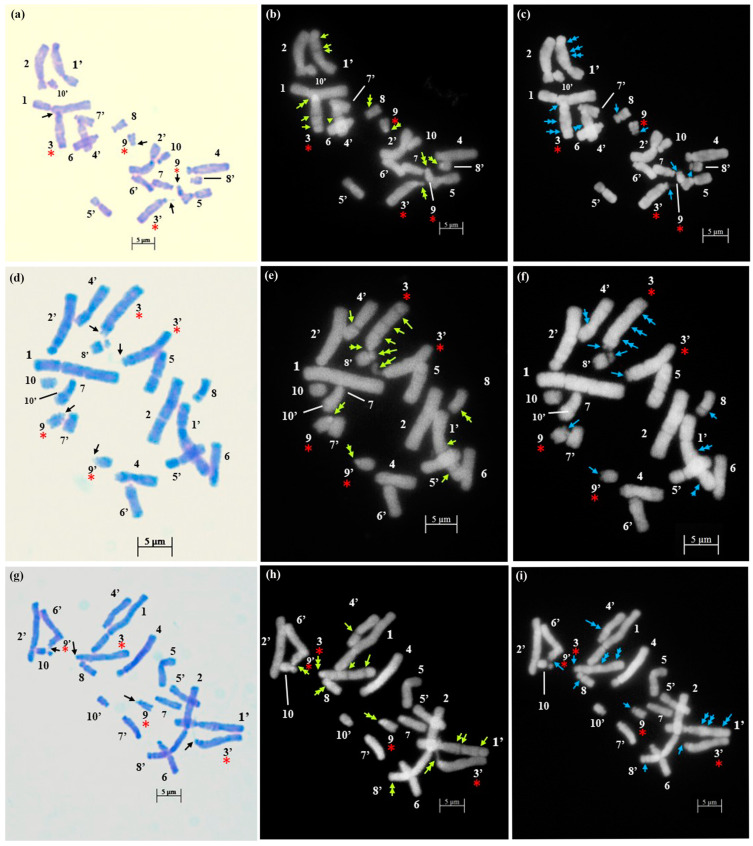
Somatic metaphase plates of *Dipcadi ursulae* Blatt. with 2n = 20 chromosomes stained with Giemsa (**a**,**d**,**g**), CMA (**b**,**e**,**h**) and DAPI (**c**,**f**,**i**). Black arrows indicate position of secondary constrictions in Giemsa-stained plates (**a**,**d**,**g**). Double and single yellow arrows indicate bands of CMA^+ve^ signals and CMA^−ve^ gaps (**b**,**e**,**h**). Double blue arrows indicate DAPI^+ve^ signals and single blue arrows mark the DAPI^−ve^ gaps (**c**,**f**,**i**). Red asterisks indicate chromosomes with secondary constrictions in all the plates. Bars 5 µm.

**Figure 5:**
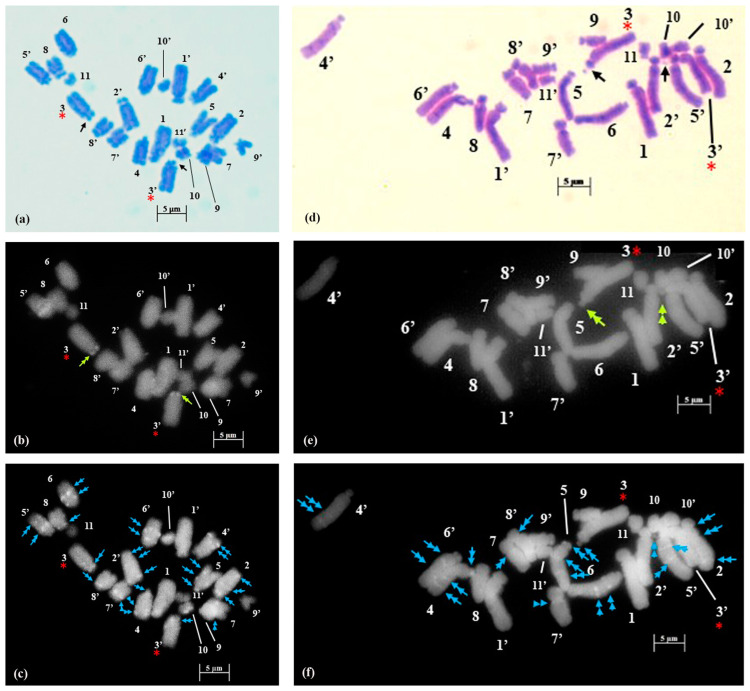
Somatic metaphase plates of *Dipcadi erythraeum* Webb & Berthel. with 2n = 22 chromosomes stained with Giemsa (**a**,**d**), CMA (**b**,**e**) and DAPI (**c**,**f**). Black arrows indicate position of secondary constrictions in Giemsa-stained plates (**a**,**d**). Double yellow arrows indicate bands of CMA^+ve^ signals in CMA-stained plates (**b**,**e**). Double blue arrows showing clear and bright DAPI^+ve^ signals (**c**,**f**). Red asterisks in all the three stained plates indicate chromosomes with secondary constrictions and an associated unusual CMA^+ve^/DAPI^0^ signal. Bars 5 µm.

The technical standardization of the methodology for EMA-based Giemsa staining allowed us to identify very clearly the numbers and the position of constrictions (primary and secondary) for the first time in all five species. The chromosomes have been categorized into three basic types [39,40] in five species: sub-median (sm), sub-terminal (st) and terminal (t). The longest chromosome pair in all the species is either with sub-terminal constriction (*D. concanense, D. goaense* and *D. erythraeum*) or with sub-median constriction (in *D. montanum* and *D. ursulae*). The smallest chromosome pair was sub-median in all the species except in *D. erythraeum*. Chromosomes with secondary constriction (i.e., chromosomes with two constrictions) have been identified for the first time in *Dipcadi* species in this study (Figure 1a, Figure 2a, Figure 3a, Figure 4a and Figure 5a). One pair of chromosomes with two constrictions was clearly identified in *D. concanense, D. goaense* and *D. erythraeum*, (Table 2, Figure 1a, Figure 2a and Figure 5a) whereas two pairs of chromosomes with secondary constrictions were identified in *D. montanum* and *D. ursulae* (Table 2, Figure 3a and Figure 4a). These were located on the 3rd pair of chromosomes in *D. concanense*, and *D. erythraeum* and on the 2nd pair in *D. goaense*. On the other hand, in *D. montanum*, the 9th and 10th pair of chromosomes were with two constrictions. In *D*. *ursulae*, these were located on the 3rd and 9th pair of chromosomes as shown in the karyotype of each species (Table 2). *Dipcadi concanense* and *D. goaense* with the same chromosome number (2n = 12), showed similar karyotype (4st + 6sm + 2st.t). Additionally, in *D*. *concanense* and *D. goaense*, the secondary constricted chromosomes were of the same type, i.e., of the two constrictions, one is subterminal (st) and the other is terminal (t), at two ends of the long arm. *Dipcadi montanum* and *D. ursulae*, with the same chromosome number (2n = 20), differ in their karyotype (Table 2) and the type of secondary constricted chromosomes. In *D. montanum*, of the two constrictions in the 9th and 10th pair of chromosomes, one is sub-median (sm) in position and the other is terminal (t) at the distal end of the short arm. In *D. ursulae*, one is sub-terminal (st) and the other is terminal (t), at the distal end of the short arm in the 3rd and 9th pair of chromosomes. The two populations of *D. erythraeum* (2n = 22) exhibited distinctly different karyotypes from all other four species. However, the type of secondary constricted chromosome was similar to *D. ursulae* (Table 2).

The interspecific and intraspecific variation in total chromatin length (TCL) was determined for all populations (Table 1). Inter-specific variation in TCL was observed between all species and between populations of *D. montanum*, *D. ursulae, D. erythraeum* (Table 1). The range of chromosome size found in these species indicated the bimodal nature of their karyotype (Table 2). The Average Chromosome Length (ACL) of the different populations in five species ranged between 5.10–5.15 µm (*D. erythraeum*) and 7.15–7.18 µm (*D. concanense*). The karyotypes of all species studied, were asymmetrical considering the centromeric position and chromosome size variation.

### 2.2. Fluorochrome Banding Pattern

**Figure 6:**
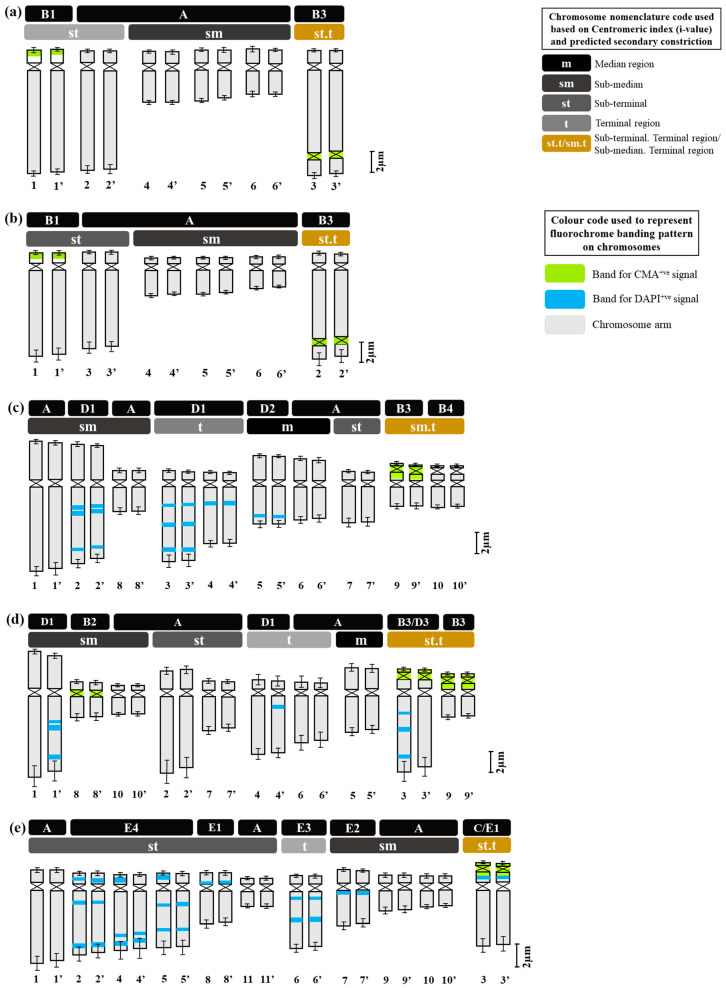
Comparative ideograms of the five species of *Dipcadi* ((**a**) *D. concanense*, (**b**) *D. goaense, (***c**) *D. montanum*, (**d**) *D. ursulae*, (**e**) *D. erythraeum)* showing CMA^+ve^ and DAPI^+ve^ banding patterns. (The upper two bars of each ideogram mention the types of CMA^+ve^ and DAPI^+ve^ signals observed on chromosome arms and category of chromosomes based on centromeric index, respectively. The numbers in the lower panel represent the numerical sequence of the chromosomes in the karyotype based on their average absolute length. Bar scale: 2 µm). Colour code used for centromeric index based chromosomal nomenclature and CMA^+ve^ and DAPI^+ve^ signals (or, bands) are described in the top-right corner of the figure.

Fluorescent banding with DAPI and CMA led to a diversified, scorable and species-specific fluorescent banding pattern in species of *Dipcadi*. 0.1 mg mL^−1^ of CMA solution required 60 min. to induce scorable bands, while, 0.1 µg mL^−1^ of DAPI solution took 25 min to induce clearly visible bands. A minimum of 15 metaphase plates for each species stained with DAPI and CMA were considered for the analysis of band patterns (Figure 1, Figure 2, Figure 3, Figure 4, Figure 5 and Figure 6). Considering the high preferential nature of CMA and DAPI in GC- and AT-rich sequences, as suggested by Barros e Silva and Guerra [41], we identified different types of heterochromatic signals/bands as GC-rich (CMA^+ve^/DAPI^−ve^), AT-rich (DAPI^+ve^/CMA^−ve^) or as AT/GC-neutral (DAPI^0^/CMA^0^) in different species of *Dipcadi* (Table 3). In chromosomes showing CMA^+ve^/DAPI^−ve^ banding pattern (type B, Table 3), DAPI staining resulted in a clear gap (DAPI^−ve^ bands) corresponding to the CMA^+ve^ signal/band. On the other hand, in chromosomes showing DAPI^+ve^/CMA^−ve^ banding pattern (type D, Table 3), CMA staining resulted in a clear gap (CMA^−ve^ bands) corresponding to the DAPI^+ve^ signal/band.

In *D. concanense and D. goaense* with 2n = 12, only four CMA^+ve^/DAPI^−ve^ bands (type B, Table 3 and Table 4, Figure 1, Figure 2 and Figure 6) were located on two pairs of chromosomes. It is noteworthy that no DAPI^+ve^ bands were identified in these two species. In *D. montanum* and *D. ursulae* (2n = 20), four and six CMA^+ve^/DAPI^−ve^ bands (type B, Table 3 and Table 4, Figure 3 and Figure 4) were observed, respectively. The distinctive feature of both species was the presence of DAPI^+ve^/CMA^−ve^ bands (type D, Table 3, Figure 3, Figure 4 and Figure 6) on the chromosomes. Sixteen type D bands were located on four pairs of chromosomes in *D. montanum* while seven bands on three chromosomes were located in *D. ursulae* (Table 4). *Dipcadi erythraeum* (2n = 22), differed from all other species in the banding type, showing two CMA^+ve^/DAPI^0^ bands (type C, Table 3 and Table 4, Figure 5) and 26 DAPI^+ve^/CMA^0^ bands (type E, Table 3 and Table 4, Figure 5 and Figure 6). The DAPI^+ve^/CMA^0^ bands were interstitial in position, located on the short arm (type E1) or long arm (E3) or on both arms (E4) of chromosomes. Thus, the fluorochrome karyotype showed significant differences in the type of bands and in the number of bands between the five species. The fluorochrome karyotypes of five species reveal similarities and distinct differences between species (Figure 6).

## 3. Discussion

Although traditional karyotype analysis can be considered obsolete in the genomic era, it is in fact quite contrary as basic karyotype information, chromosome number, genome size, and position of landmarks including repetitive DNA, will remain important for necessary data interpretation [42,43,44,45,46,47,48]. Chromosome features used in cytotaxonomy may present a continuous variation (average chromosome length, mean arm ratio or r index, symmetry indices) or a discontinuous variation (chromosome number, heterochromatic bands, number of rDNA sites) [46,49]. Measurements of chromosome arms for the identification of chromosomes in a karyotype are useful to quantify differences or similarities among karyotypes. The symbols used in the present study to describe karyotypes correspond to those coined by Levan et al. [39], as described by Mitrenina et al. [40]. Although most publications in plant cytogenetics followed the nomenclature for chromosome morphology as reviewed by Levan et al. [39], some variations in nomenclature [50] and development of R scripts for the determination of standardized karyotype have also been reported [45,48].

In this study, we have presented a karyotype analysis of five endemics, threatened Indian species of *Dipcadi*, which was reported as a disappearing genus in India [51]. *Dipcadi* with 41 species distributed in the Mediterranean region, Africa and Southeast Asia [13,20], is poorly defined taxonomically because of overlapping morphological characters [12]. Consistent with the general trend in Scilloideae, continuous variation is prevalent in most Indian taxa requiring detailed taxonomic characterization, including molecular and cytogenetic characterization. Molecular phylogenetic studies in the tribe Ornithogaloideae (Subfamily Scilloideae) recognized nineteen monophyletic genera, including *Dipcadi* [5].

Somatic chromosome counts available globally for about 14 species of *Dipcadi* [25] have revealed wide diversity in chromosome number (2n = 6, 8, 16, 20, 22, 24, 32, 40). Since *D. serotinum*, with a broad distribution (Europe and Northern Africa to the Arabian Peninsula and India) shows 2n = 2x = 8 chromosomes with n = 4 [33], a base chromosome number [49,52] of x = 4 has been suggested [30]. Thus, considering x = 4, taxa with 2n = 12, 20, 22 in the present study may be polyploid derivatives. However, the probable base chromosome number of x = 6 has also been proposed for the genus *Dipcadi* [26] according to which in the present study, species with 2n = 12 are diploids (*D. concanense* and *D. goaense*) while species with 2n = 20, 2n = 22 (*D. ursulae*, *D. montanum, D. erythraeum*) presumably resulted from descending dysploidy and subsequent polyploidisation, and are thus probable hypotetraploids.

In the present study, the somatic chromosome number of 2n = 12 was observed in *D. concanense*, and *D. goaense* reconfirms earlier chromosome number reports in the two species [19,28,29]. Chromosome numbers of *D. montanum* and *D. ursulae* were observed to be 2n = 20, while *D. erythraeum* showed 2n = 22. Although Mahabale and Cheenavariah [27] reported 2n=20 for *D. montanum*, Naik [32] reported two cytological races for *D. montanum* collected from Aurangabad showing 2n = 10 and 2n = 12 in somatic metaphases. In this study, we reconfirm 2n = 20 for *D. montanum*. Jakhi et al. [31] first reported 2n = 22 and n = 11 in *D. erythraeum* collected from Rajasthan, reconfirmed by Jehan et al. [12], although 2n = 20 has also been reported in the species [30]. We confirm 2n = 22 in *D. erythraeum* in two populations from Rajasthan.

Meiotic analysis in species reported revealed regular bivalent formation in pollen mother cells [14,30,31,32,33]. However, in some species, low pollen fertility [31], the occurrence of univalents [30] and abnormalities during meiosis have also been reported [33]. Hybridization followed by polyploidisation may have resulted in ascending dysploid series of chromosome numbers in the genus *Dipcadi*. Dysploid variation is caused by complex mechanisms [52] and further analyses in a large number of species are a prerequisite to suggest the trend of karyotype evolution in the genus *Dipcadi*.

Previous reports of karyotype analysis on Indian species of *Dipcadi* are few and have not revealed distinct morphology of the karyotype with respective to a position of centromere and secondary constriction. In *D. erythraeum*, Jakhi et al. [31] described chromosome morphology revealing one pair of long and two pairs of short chromosomes with sub-terminal constrictions, while the remaining chromosomes were sub-median. Rawat et al. [30] determined the karyotype formula revealing the majority of telocentric chromosomes followed by sub-metacentrics. They also stated that the analysis of chromosomes with secondary constrictions could not be obtained due to technical difficulties. The karyotype of *D. goaense* was reported to be similar to *D. concanense* [14,27]. 

This is the first report of EMA-Giemsa-based karyotype analysis establishing the modal karyotypes of five endemic or threatened Indian species of *Dipcadi* showing the presence of chromosomes with secondary constrictions in each species, varying in type and number. The karyotypes are characterized by the predominance of acrocentric/telocentric chromosomes with distinctly bimodal or graded chromosome complement. 

In most plant species, the centromere or primary constriction is present in all the chromosomes, while on some chromosomes, a secondary constriction at the nucleolar organizer region (NOR) has been identified from the earliest microscopy [53,54]. At metaphase, NORs are often visible as secondary constrictions as the arrays of genes active at the previous metaphase remain decondensed [54]. Chromosomes with secondary constrictions are considered landmark chromosomes in karyotype analysis. In *D. concanense*, *D. goaense* and *D. erythraeum* one pair of chromosomes with secondary constrictions were identified while in *D. montanum* and *D. ursulae*, two pairs of chromosomes with secondary constrictions were identified.

The EMA-based Giemsa-stained karyotypes of two populations of *D. concanense and D. goaense* (from type locality), were similar in number and morphology including the type of nucleolar chromosomes. On the other hand, *D. montanum*, *D. ursulae* and *D. erythraeum* differ distinctly in their karyotypes including the number and type of nucleolar chromosomes. Fluorescent banding, particularly with CMA and DAPI, has been frequently used in a wide range of plant species to characterize individual chromosomes and delineate heterochromatic regions comprised of repetitive DNA sequences at different locations in a chromosome [36,55]. Chromosomal CMA^+ve^ bands imply the prevalence of heterochromatic GC elements mainly surrounding the NORs [55,56] whereas the DAPI^+ve^ bands reflect a type of condensed heterochromatin occupied by AT elements since DAPI is specific for AT-rich DNA stretches [36,57]. The same fluorochromes may also negatively stain AT-poor (DAPI^−ve^) or GC-poor (CMA^−ve^) heterochromatin blocks [55]. 

It is apparent from the fluorochrome band profiles in the present study that all five species of *Dipcadi* exhibited the CMA^+ve^ band in one of the constriction regions or terminal satellites of the nucleolar chromosome. These CMA^+ve^ bands were DAPI^−ve^, showing a clear gap corresponding to the CMA^+ve^ bands in all species except in *D. erythraeum*. It is noteworthy that by using fluorochrome banding with base-specific fluorochromes [42], GC-rich heterochromatin has been identified in all species of *Dipcadi*, mostly in the nucleolar chromosomes. The majority of heterochromatic bands [42] have been reported to be AT-rich and are usually at interstitial regions in species with medium and large chromosomes. 35S rRNA genes often have been found to coincide with GC-rich bands [55]. The distal CMA^+ve^/DAPI^−ve^ bands in the short arm of long chromosomes in *D. concanence* and *D. goaense* has not been observed in any other species in the present study. The CMA^+ve^ signals are generally considered to represent GC-rich heterochromatin found mostly at NORs and also at proximal positions, coinciding with DAPI-negativity in the majority of plants reported [55]. It is now known that secondary constrictions represent only the expression of rRNA genes that were active during the last interphase [58]. Other functional sites may not form secondary constrictions if located at the terminal end of chromosomes [59].

AT-specific DAPI^+ve^ banding profile revealed unique species-specific characteristic features for the first time in *Dipcadi*. DAPI^+ve^ bands were distributed in the different interstitial regions of the long arm and short arm of the chromosomes in all species except in *D. concanence* and *D. goaense*. No DAPI^+ve^/CMA^−ve^ or DAPI^+ve^/CMA^0^ signals/bands were obtained in *D. concanence* and *D. goaense. Dipcadi montanum* and *D. ursulae* showed distinctive DAPI^+ve^/CMA^−ve^ bands in the chromosomes of the diploid complement (2n = 20). However, the two species differ in the number and occurrence of DAPI^+ve^/CMA^−ve^ bands in the homologous chromosomes. In *D. montanum*, sixteen DAPI^+ve^/CMA^−ve^ bands occur in four chromosome pairs. While in *D. ursulae*, seven DAPI^+ve^/CMA^−ve^ bands were observed in three chromosomes, but not in the corresponding homologous pair. Thus, in *D. ursulae*, three out of ten pair of chromosomes show heteromorphism in homologous chromosomes with respect to DAPI^+ve^/CMA^−ve^ banding patterns.

*Dipcadi erythraeum* with 2n = 22, shows distinct variation in banding type and pattern from all other species of *Dipcadi* studied. CMA^+ve^/DAPI^0^ signals in one of the constriction regions (in the chromosome pair with secondary constriction or nucleolar chromosome) extended to the short arm and terminal satellite. No CMA^+ve^/DAPI^−ve^ bands were observed in any of the chromosomes. DAPI^+ve^/CMA^0^ signals were observed in seven chromosome pairs including one pair of nucleolar chromosomes. A total of 26 DAPI^+ve^/CMA^0^ bands were found on the seven chromosomes. Thus, in *D. erythraeum* two out of seven pair of chromosomes show heteromorphism in homologous chromosomes. Rawat et al. [30] suggested an amphidiploid origin for *D. erythraeum*. Jehan et al. [12] based on studies using molecular markers found *D. erythraeum* from Rajasthan to be distinctly different from the other species of Western Ghats. The heteromorphism in the banding pattern (DAPI^+ve^) supports both studies. Thus, in *D. ursulae* and *D. erythraeum*, CMA^+ve^/DAPI^+ve^ signals in different regions of the same chromosome indicate the heterochromatin variation acquired by the species. The distribution of CMA and DAPI bands is a reflection of heterochromatin composition and variations acquired by different species [60,61,62]. AT-specific DAPI^+ve^ banding pattern obtained in the present study revealed unique species-specific characteristic features for the first time.

In the subfamily Scilloideae, fluorochrome banding and fluorescence in situ hybridization (FISH) has been reported in some species under the tribe Hyacintheae. In *Bellevalia*, CMA^+ve^ signals were associated with nucleolar chromosomes and rDNA probes colocalized with CMA^+ve^ signals [63], although some variations have been reported in *B. romana* [64]. In *Muscari*, with bimodal karyotype, CMA^+ve^ signals were located at NOR and rDNA probes colocalized with CMA^+ve^ signals [65,66]. Interspecific variation in the distribution of DAPI^+ve^ signals [65,67] and CMA^+ve^ signals [67] have been reported in *Muscari*. Varied distribution of DAPI^+ve^ signals has also been reported in species of *Lachenalia* [68,69]. In *Drimia* (tribe Urgineeae), CMA^+ve^/DAPI^−ve^ signals were associated with nucleolar chromosomes, with some interspecific variations in additional signals [37]. In *Albuca bracteata*, (tribe Ornithogaleae), CMA^+ve^ signals were detected at NOR regions, colocalized with rDNA signals while DAPI^+ve^ signals were also reported at the centromeric or intercalary regions in the species [70].

Deshpande et al. [20] investigated the phylogenetic relationship between the two endemic and critically endangered Indian species of *Dipcadi*, *D. concanense* and *D. goaense*, using a plastid (*mat*K) and ITS sequences. This study [20] revealed that *D. concanense* and *D. goaense*, were not only morphologically similar [19], with the same chromosome number [14], but they were also phylogenetically closely related species. In the present study, the karyotype analysis based on chromosome morphometric data as well as the fluorochrome banding pattern of *D. concanense* and *D. goaense*, were found to be very similar, and in agreement with findings based on molecular phylogenetic data [20]. Jehan et al. [12] studied in detail genetic diversity among the three genera, *Drimia*, *Dipcadi* and *Ledebouria* of subfamily Scilloideaea in India, using RAPD and SRAP markers. The study resolved the three genera into monophyletic groups corresponding to three subfamilies (now subtribes); *Urginoideae*, *Hyacinthoideae* and *Ornithogaloideae*. Among the Indian species of *Dipcadi* (excluding *D. goaense*), studied by Jehan et al. [12], *D. concanense* was found to be very distinct from other species of Western Ghats and *D. erythraeum* was also found to be a genetically distinct species in this study. The species from the Western Ghats formed a well distinct group, “whereas, northern Indian species, *D. erythraeum* from Rajasthan and *Dipcadi serotinum* from Delhi stood out as well differentiated taxa”. Jehan et al. [12] suggested that *Dipcadi serotinum* may have been introduced from Europe, as the flowering time differs from the Indian species. Thus, the unique fluorochrome banding patterns of *D. montanum* and *D. ursulae*, reveal that though the two species share the same chromosome number, the species are distinctly different as observed by Jehan et al. [12].

The application of fluorescent banding for comparative analysis of karyotypes has enriched present-day cytogenetics enormously as an integrative approach to solving the issues of systematics and phylogeny [71,72,73]. The present EMA-based Giemsa and fluorescent banding karyotype in five Indian *Dipcadi* species have confirmed distinct patterns and diversity of landmark nucleolar chromosomes. The result has revealed a diverse number of species-specific AT-rich, DAPI-positive repetitive sequences (0–26 in number) for the first time in the genus *Dipcadi*. These may be considered useful molecular markers for analyzing genetic diversity and studying genome evolution in other species. To resolve the interspecific phylogenetic and evolutionary relationships more molecular cytogenetic-based chromosome analysis using fluorescent banding and fluorescence in situ hybridization (FISH) deserves attention.

## 4. Materials and Methods

### 4.1. Plant Materials and Their Collection

Collection of different populations of *Dipcadi* species was possible as part of this study under the guidance of Professor SR Yadav and Dr. MM Lekhak, Shivaji University, Kolhapur, Maharashtra and with the help of Professor NS Shekhawat, Jodhpur University, Rajasthan. We could not collect/obtain any other Indian species for this study. Herbarium vouchers were prepared for each species, identified and deposited to the Shivaji University Herbarium, Kolhapur as well as Calcutta University Herbarium. Brief information on the place of collection of these five species is given in Table 1. Bulbs of each species were grown in pots and maintained in the experimental garden of the Department of Botany, University of Calcutta.

### 4.2. Mitotic Chromosome Preparation and Giemsa Staining

Ten to fifteen actively growing root tips from bulbs of each species were harvested during the months of June to August and pre-treated with 0.5% colchicine for 4 to 4.5 h at 14–16 °C [74]. The pre-treated root tips were fixed in a 3:1 methanol-acetic acid solution overnight and stored at −20 °C. The chromosome preparations were performed through standardization of the basic EMA technique following our earlier protocol [37,74] with modifications required. Fixed root tips were placed in water and kept at 4 °C for 3 h. One to two mm root tips were excised and carefully placed inside a microtube containing a cocktail enzyme mixture containing 0.15% Pectolyase (Y-23) plus 0.75% Macerozyme (R-10) and 1% Cellulose (Onozuka RS) along with 1mM EDTA. Root tips were incubated at 37 °C for 80–90 min. Enzyme-digested root tips were washed with distilled water and macerated in freshly prepared acetic methanol (1:3) solution on glass slides. Air-dried slides were stained in 2% Giemsa solution (Giemsa azure eosin methylene blue solution, Merck, Darmstadt, Germany) in 1/15th phosphate buffer (2.390 g Na_2_HPO_4_ and 0.900 g KH_2_PO_4_ in 100 mL distilled water) for 20–25 min at room temperature. A staining period of 25 min with Giemsa was found optimum for *Dipcadi* species. Giemsa-stained slides were screened under Axio Lab. A1 Carl Zeiss microscope to assess the quality of cytological preparations. Data sheets for individual species were prepared for selected well-scattered metaphase plates. Photomicrographs were taken under Axio Lab. A1 microscope fitted with a CCD camera and computer.

### 4.3. Karyotype Analysis

Somatic chromosome numbers and karyotypes were determined from 20–25 well-scattered Giemsa-stained metaphase plates of each population of species. The software Axiovision L.E 4 (Carl Zeiss, Jena, Germany), was used for chromosome morphometric data. A minimum of 10 well-scattered metaphase plates were selected for each population/species and analysed using this software for the estimation of short arm length (s), long arm length (l), arm ratios (r = l/s), chromosome length (CL), and total chromosome length (TCL). The centromeric index (i-value) was determined following Levan et al. [39] and Mitrenina et al. [40]. Absolute and relative chromosome lengths were calculated [75]. Karyotype formulae and the respective ideogram for each of the individual species were generated using the chromosome morphometric data [76].

### 4.4. Fluorochrome Staining of Somatic Chromosomes

Giemsa-stained slides were de-stained with 70% methanol for 45 min, air dried and further used twice for two separate fluorochrome staining with 4′-6-Diamidino-2-phenylindole (DAPI) and Chromomycin A_3_ (CMA) following the protocol described earlier [74,76], with minor modifications. Slides were incubated in McIlvaine buffer I (0.1 M citric acid, 0.2 M Na_2_HPO_4_, pH 7.0) for 30 min and stained with 0.1 µg/mL DAPI solution for 25–30 min. Slides were washed in the same buffer and counterstained with Actinomycin D (0.25 mg/mL) for 15 min. Slides were air-dried and mounted in non-fluorescent glycerol. The slides were kept overnight at 4 °C for maturation and were examined under the microscope with a UV filter cassette and images were captured with a CCD camera. For CMA staining, the slides were de-stained in 70% Methanol and air-dried. Slides were incubated in McIlvaine buffer I (0.1 M citric acid, 0.2 M Na_2_HPO_4_, pH 7.0) for 30 min and then in McIlvaine buffer II (with 5 mM MgCl_2_·6H_2_O) for 15 min. The slides were then flooded with 0.1 mg/mL CMA for 55–70 min. Excess stain was washed off in McIlvaine buffer II, air dried and mounted in non-fluorescent glycerol and kept at 4 °C refrigerator for 48 hrs. The observations were made by fluorescence microscopy with a BV filter cassette and the images were captured with a CCD camera.

### 4.5. Statistical Analysis

Data from at least ten different scattered metaphase plates were taken for determination of all the karyo-morphometric data, and data for each karyo-morphometric parameter was expressed as the mean ± standard deviation (SD). One-way analysis of variance (ANOVA) was used to analyse the data in order to find statistically significant variations in the mean values among the five species of *Dipcadi*. Using SPSS statistic software (IBM^®^, Armand, NY, USA) version 17.0, a descriptive post hoc mean separation analysis for the karyo-morphometric data set was carried out using Duncan’s multiple range test (DMRT) at the 5% probability level.

## Figures and Tables

**Table 1:** Collection details, somatic chromosome number and total chromosome Length (TCL) in different populations of Indian species of *Dipcadi*.

Species	Population	Site of Collection	Geographic Details	Chromosome Number (2n)	TCL (Mean ± µm) *
*D. concanense*	Dcon1	Rajapur, Maharashtra	16.6571° N, 73.5211° E	12	85.68 ± 0.53 ^b^
	Dcon2	Ratnagiri, Maharashtra	16.9902° N, 73.3120° E	12	86.20 ± 0.71 ^b^
*D. goaense*	Dgoa1	Quepem District, South Goa	15.2282° N, 74.0647° E	12	81.98 ± 0.72 ^a^
*D. ursulae*	Durs1	Thosegar, Maharashtra	17.6031° N, 73.8478° E	20	110.58 ± 1.38 ^c^
	Durs2	Panhala, Maharashtra	16.8107° N, 74.1181° E	20	117.00 ± 0.87 ^f^
	Durs3	Satara, Maharashtra	17.6805° N, 74.0183° E	20	118.08 ± 1.00 ^g^
*D. montanum*	Dmon1	Ajara, Maharashtra	16.1159° N, 74.2106° E	20	118.84 ± 0.43 ^g^
	Dmon2	Badami, Karnataka	15.9186° N, 75.6761° E	20	112.00 ± 0.64 ^d^
*D. erythraeum*	Dery1	Jaisalmer, Rajasthan	26.9157° N, 70.9083° E	22	112.24 ± 0.43 ^d^
	Dery2	Jodhpur, Rajasthan	26.2389° N, 73.0243° E	22	113.30 ± 0.98 ^e^

* Values followed by same letter are not significantly different; according to Duncan’s test (*p* = 0.05).

**Table 2:** Chromosome morphometric data generated through EMA-Giemsa staining in the five Indian species of *Dipcadi*.

Species & Population		Absolute Length of Longest Chromosome (Mean ± SD in µm) *	Absolute Length of Shortest Chromosome (Mean ± SD in µm) *	ACL (Mean ± SD in µm) *	No. of SAT Chromosome & Ordering No. of SAT Bearing Pair	Diploid Karyotype Formula	Diagrammatic Representation of Karyotype (Haploid Set)
*D. concanense* (Dalzell) Baker 2n = 12	Dcon1	11.25 ± 0.30 ^b^	3.10 ± 0.20 ^u^	7.15 ± 0.40 ^z^	2 (3rd pair)	4st + 6sm + 2st.t	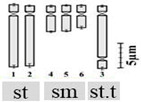
	Dcon2	11.14 ± 0.45 ^b^	3.19 ± 0.14 ^u^	7.18 ± 0.31 ^z^			
*D. goaense* Prabhug. 2n = 12	Dgoa1	10.93 ± 1.36 ^b^	3.05 ± 0.47 ^u^	6.83 ± 0.81 ^yz^	2 (2nd pair)	4st + 6sm + 2st.t	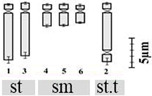
*D. montanum* (Dalzell) Baker 2n = 20	Dmon1	11.79 ± 0.87 ^b^	2.35 ± 0.18 ^st^	5.94 ± 0.55 ^xy^	4 (9th & 10th pair)	6sm + 4t + 4m + 2st + 4sm.t	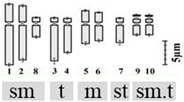
	Dmon2	11.66 ± 0.68 ^b^	2.40 ± 0.15 ^t^	5.60 ± 0.54 ^x^			
*D. ursulae* Blatt. 2n = 20	Durs1	11.29 ± 1.66 ^b^	2.01 ± 0.29 ^qr^	5.52 ± 0.86 ^x^	4 (3rd & 9th pair)	6sm + 4st + 4t + 2m + 4st.t	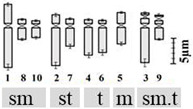
	Durs2	11.54 ± 0.32 ^b^	2.05 ± 0.22 ^qr^	5.85 ± 0.92 ^xy^			
	Durs3	11.79 ± 0.87 ^b^	2.01 ± 0.28 ^qr^	5.90 ± 0.74 ^xy^			
*D. erythraeum* Webb & Berthel. 2n = 22	Dery1	7.88 ± 1.02 ^a^	1.66 ± 0.20 ^p^	5.10 ± 1.05 ^x^	2 (3rd pair)	12st + 2t + 6sm + 2st.t	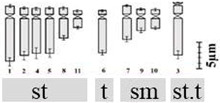
	Dery2	7.93 ± 1.58 ^a^	1.72 ± 0.16 ^pq^	5.15 ± 0.75 ^x^			

* Values followed by the same letter are not significantly different; according to Duncan’s test (*p* = 0.05).

**Table 3:** A brief typification of CMA and DAPI fluorescent bands observed in somatic chromosomes of five Indian species of *Dipcadi*.

Major Band/Signal Types Based on CMA/DAPI Staining	Sub-Types Based on Position of Bands/Signals on Chromosomes	Position of Bands/Signal(s) on Chromosome Arms	Diagrammatic Representation	Species Name
Type A CMA^0^/DAPI^0^	A	No distinct signal	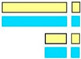	In all species
Type B CMA^+ve^/DAPI^−ve^	B1	Short arm, distal to constriction	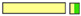	*D. concanense*, *D. goaense*
	B2	Centromeric region	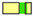	*D. ursulae*
	B3	Nucleolar	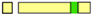	*D. concanense*, *D. goaense*
		Nucleolar, extended to terminal satellite	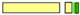	*D. ursulae*
		Nucleolar, extended to short arm and terminal satellite	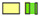	*D. montanum*, *D. ursulae*
	B4	Short arm and terminal satellite	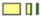	*D. montanum*
Type C CMA^+ve^/DAPI^0^	C	Nucleolar, extended to short arm and terminal satellite	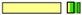	*D. erythraeum* *
Type D DAPI^+ve^/CMA^−ve^	D1	Long arm, interstitial, 1–3 in number	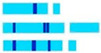	*D. montanum*, *D. ursulae*
	D2	Long arm, Distal	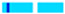	*D. montanum*
Type E DAPI^+ve^/CMA^0^	E1	Short arm, interstitial	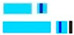	*D. erythraeum* *
	E2	Long arm, proximal to constriction	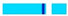	*D. erythraeum*
	E3	Long arm, interstitial, 2 in number (2 bands)	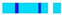	*D. erythraeum*
	E4	Both arms, interstitial, 3 in number	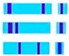	*D. erythraeum*

[Light yellow and light blue line diagrams represent CMA and DAPI-stained chromosome arms, respectively. The fluorescent green bands indicate CMA^+ve^ signals on CMA-stained chromosomes. The dark blue bands indicate DAPI^+ve^ signals on DAPI-stained chromosome arms, respectively. * Chromosome pair in *D. erythraeum* showing both CMA^+ve^ (C type) and DAPI^+ve^ (E1 type) signals].

**Table 4:** CMA and DAPI fluorescent banding patterns somatic chromosomes of five Indian species of *Dipcadi*.

Sl. No.	Species & Chromosome Number	Order of Nucleolar Pair	CMA^+ve^ Bands			DAPI^+ve^ Bands			Total No. CMA^+ve^ & DAPI^+ve^ Bands/2n	Fluorescent Karyotype (2n) *
			No.	Chromosome Pair (p)/Single (s)	Type	No.	Chromosome Pair (p)/Single (s)	Type		
1.	*D. concanense * (2n = 12)	3rd	2	1st (p)	B1	Nil	--	--	4	8A + 4B
			2	3rd (p)	B3					
2.	*D. goaense * (2n = 12)	2nd	2	1st (p)	B1	Nil	--	--	4	8A + 4B
			2	2nd (p)	B3					
3.	*D. montanum * (2n = 20)	9th 10th	2	9th (p)	B3	6	2nd (p)	D1	20	8A + 4B + 8D
			2	10th (p)	B4	6	3rd (p)	D1		
						2	4th (p)	D1		
						2	5th (p)	D2		
4.	*D. ursulae* (2n = 20)	3rd 9th	2	3rd (p)	B3	3	1st (s)	D1	13	10A + 4B + 2B/D + 4D
			2	8th (p)	B2	3	3rd (s)	D3		
			2	9th (p)	B3	1	4th (s)	D1		
5.	*D. erythraeum * (2n = 22)	3rd	2	3rd (p)	C	6	2nd (p)	E4	28	8A + 2C/E+12E
						2	3rd (p)	E1		
						4 + 1	4th (2p + 1s)	E4		
						4 + 1	5th (2p + 1s)	E4		
						4	6th (p)	E3		
						2	7th (p)	E2		
						2	8th (p)	E1		

* Type A (CMA^0^/DAPI^0^), Type B (CMA^+ve^/DAPI^−ve^), Type C (CMA^+ve^/DAPI^0^), Type D (DAPI^+ve^/CMA^−ve^), and Type E (DAPI^+ve^/CMA^0^).

## Data Availability

All data have been presented in the paper.
